# Clara cell 10 (CC10) protein attenuates allergic airway inflammation by modulating lung dendritic cell functions

**DOI:** 10.1007/s00018-024-05368-z

**Published:** 2024-07-30

**Authors:** Yu-Dong Xu, Mi Cheng, Jun-Xia Mao, Xue Zhang, Pan-Pan Shang, Jie Long, Yan-Jiao Chen, Yu Wang, Lei-Miao Yin, Yong-Qing Yang

**Affiliations:** Shanghai Research Institute of Acupuncture and Meridian, Yueyang Hospital of Integrated Traditional Chinese and Western Medicine, Shanghai University of Traditional Chinese Medicine, Shanghai, 201203 People’s Republic of China

**Keywords:** Uteroglobin, Allergic response, Immunomodulation, Respiratory diseases, Cytokine signaling, T cell differentiation, cDC2

## Abstract

Allergic asthma is a complex inflammatory disorder predominantly orchestrated by T helper 2 (Th2) lymphocytes. The anti-inflammatory protein Clara Cell 10-kDa (CC10), also known as secretoglobin family 1A member 1 (SCGB1A1), shows promise in modulating respiratory diseases. However, its precise role in asthma remains unclear. This study examines the potential of CC10 to suppress allergic asthma inflammation, specifically assessing its regulatory effects on Th2 cell responses and dendritic cells (DCs). Lower CC10 levels in asthma were observed and correlated with increased IgE and lymphocytes. *Cc10*^*−/−*^ mice exhibited exacerbated allergic airway inflammation marked by increased inflammatory cell infiltration, Th2 cytokines, serum antigen-specific IgE levels, and airway hyperresponsiveness (AHR) in house dust mite (HDM)-induced models. Conversely, recombinant CC10 significantly attenuated these inflammatory responses. Intriguingly, CC10 did not directly inhibit Th cell activation but significantly downregulated the population of CD11b^+^CD103^−^ DCs subsets in lungs of asthmatic mice and modulated the immune activation functions of DCs through NF-κB signaling pathway. The mixed lymphocyte response assay revealed that DCs mediated the suppressive effect of CC10 on Th2 cell responses. Collectively, CC10 profoundly mitigates Th2-type allergic inflammation in asthma by modulating lung DC phenotype and functions, highlighting its therapeutic potential for inflammatory airway conditions and other related immunological disorders.

**Supplementary Information:**

The online version contains supplementary material available at 10.1007/s00018-024-05368-z.

## Introduction

Allergic asthma, characterized by chronic inflammation of the airways, is predominantly driven by T helper 2 (Th2) lymphocytes. These cells release cytokines such as IL-4, IL-5, and IL-13, which contribute to the pathological changes, including increased IgE production, eosinophil activation, and airway hyperresponsiveness (AHR) [1]. Recent studies have revealed the heterogeneity and complexity of asthma, indicating that therapies targeting Th2 cytokines benefit only a subset of patients [2]. This suggests that strategies targeting upstream immunoregulatory cells and mediators in antigen presentation might more effectively regulate the onset and progression of Th2-mediated airway inflammation.

Emerging evidence highlights the critical role of airway epithelial cells, the first line of defense against respiratory pathogens, in initiating inflammatory responses to allergens. These cells produce active mediators and cytokines that guide dendritic cells (DCs) toward an aberrant Th2 immune response [3, 4]. In turn, Th2 cytokines such as IL-4 induce a range of gene expressions in airway epithelial cells, including pro-inflammatory and remodeling genes, thereby establishing a Th2-polarized condition in the airway [5]. Notably, mucosal non-ciliated airway epithelial cells, also known as Clara cells or Club cells, which are predominantly found in the respiratory epithelium of terminal bronchioles, along with their secretory products, are reported to play a protective role in maintaining airway homeostasis against environmental allergens like house dust mites (HDM) [6–8].

Clara cell 10-kDa protein (CC10), or Uteroglobin/Clara cell secretory protein (CCSP)/secretoglobin family 1A member 1 (SCGB1A1), is a major secretory product of Clara cells and abundant in the peripheral airways [7]. It functions as an anti-inflammatory and immunosuppressive protein through inhibiting phospholipase A2 (PLA2) [9], Th2 cell differentiation [10], and NF-κB signaling [11]. Abnormal CC10 levels are observed in various respiratory conditions, including asthma [12], COPD [13], and cystic fibrosis lung disease [14]. Clinical studies have demonstrated a reduced expression of CC10 in asthmatic patients compared to healthy individuals [12, 15, 16]. Conversely, CC10 expression in the airways significantly increases following allergen-specific immunotherapy, concomitant with the reduction of local Th2 cytokines, underscoring its role as a pivotal endogenous regulator of airway inflammation [17]. However, the potential of CC10 as a therapeutic agent for Th2-predominant inflammation in allergic asthma and its mechanism of action on airway Th2 cells remain to be clarified.

Allergen exposure can also activate pulmonary DCs beneath the epithelial barrier of airways [18]. These activated DCs express MHC-II, co-stimulatory molecules (CD80, CD86, CD40), and secrete pro-inflammatory cytokines (IL-6, TNF-α), activating naïve and memory T cells [19, 20]. Meanwhile, the cytokine micromilieu of airway epithelium influences the differentiation of pulmonary DCs, with CD11b^+^ DCs playing a critical role in initiating Th2 immunity to HDM allergen [21, 22]. Considering the intimate interaction between airway epithelial cells and DCs, CC10 may directly affect pulmonary DCs, altering Th2 activation and mitigating allergic airway inflammation in asthma.

In this study, we generated CC10 gene-deficient (*Cc10*^*−/−*^) mice, and then utilized a house dust mite (HDM)-induced murine asthma model and cultured bone marrow-derived dendritic cells (BMDCs), to evaluate the role of CC10 in alleviating Th2-mediated airway inflammation in asthma and its effects on the differentiation phenotype and immune activation functions of lung DCs and BMDCs. Additionally, we investigated whether CC10 directly suppresses Th cell activation, and further used mixed lymphocyte reaction assays to study the crucial role of DCs in the suppression of Th2 cell responses exerted by CC10.

## Materials and methods

### Measurement of CC10 expression in human peripheral blood plasma

This study included asthmatic patients (*n* = 20; mean age, 39 ± 11 years) enrolled in Protocol NCT 01931696, sanctioned by the Human Research Ethics Committee of Yue Yang Hospital, Shanghai University of Traditional Chinese Medicine (Approval number: 2013–041), and healthy controls (*n* = 20; mean age, 33 ± 9 years). The asthmatic participants met the Global Initiative for Asthma (GINA) diagnostic criteria and demonstrated a forced expiratory volume in 1 s (FEV1) increase of 200 mL and 12% above baseline. Informed consent was obtained from all participants upon admission. Peripheral blood samples were collected in 10 mL sodium heparinized Vacutainers and were then centrifuged at 800 g for 10 min at 4ºC to separate plasma. Plasma CC10 concentrations were determined using an enzyme-linked immunosorbent (ELISA) assay kit (Cat: DUGB00, R&D Systems, Minneapolis, MN, USA), following the manufacturer's instructions.

### Mice

C57BL/6 J mice were acquired from Beijing Charles River Experimental Animal Technology Center (SCXK (Shanghai) 2019–0001, Shanghai, China). OVA (ovalbumin)-specific TCR transgenic mice OT-II (B6.Cg-Tg(TcraTcrb)425Cbn/J; 004194) were originally from the Jackson Laboratory. CC10 deficient (*Cc10*^*−/−*^) mice were generated by Shanghai Model Organisms Center using CRISPR/Cas9 technology on a C57BL/6 J mouse background. *Cc10*^*−/−*^ mice were verified via polymerase chain reaction (PCR) and western blot. The Institutional Animal Care and Use Committee of Shanghai University of Traditional Chinese Medicine approved the experiments, which included all mice aged 8–10 weeks and maintained under specific pathogen-free conditions, in line with the Guide for the Care and Use of Laboratory Animals.

### Allergen-induced mouse asthma model and treatment protocol

Mice were anesthetized with 2% isoflurane and sensitized intranasally with 10 µg HDM extracts (Greer Laboratories, Lenoir, NC) in 10 µL sterile PBS on day 0 and day 7, followed by five consecutive daily challenges (day 14–18) with 20 µg HDM extracts. 48 h post-final challenge, lung function was assessed under anesthesia, and mice were then euthanized for evaluation of allergic airway inflammation markers. Control mice received the same volume of PBS. Mice in the CC10 treatment group received 25 µg of recombinant murine CC10 (PrimeGene, Cat: 622–30, Shanghai, China) intranasally 2 h prior to each HDM exposure, whereas the vehicle control group received an equivalent volume of PBS.

### Airway responsiveness to methacholine

Two days post-final HDM challenge, mice were anesthetized with 1% pentobarbital sodium (10 mL/kg), tracheotomized, and intubated with an 18-gauge catheter connected to the FinePointe resistance and compliance system (Data Sciences International, Inc., St. Paul, MN, USA). Mechanical ventilation was set at 140 breaths/min with a tidal volume of 0.2 mL and a positive end-expiratory pressure of 2 cm H_2_O. Following a 5-min stabilization period, the mice received exposure to aerosolized PBS (baseline). Subsequently, they were subjected to escalating doses of methacholine (MCh) (1.5, 3, 6, and 12 mg/mL; Sigma-Aldrich). Lung resistance (R_L_) values, recorded for 3 min post each Mch challenge, were expressed as deviations from baseline PBS responses.

### Tracheal ring contraction assay

Tracheal rings were harvested from 6 to 8-week-old sex-matched mice as previously described [23]. Tracheal segments were incubated with CC10 (1 µg/mL), either alone or in combination with IL-13 (100 ng/mL), for 12 h before acetylcholine (ACh) stimulation. The segments were then suspended in organ bath chambers (Panlab Harvard Apparatus, Holliston, Massachusetts, USA) containing oxygenated Krebs solution (pH 7.4) at 37 °C. After a 30-min equilibration at a resting tension of 1 g, tracheal rings were subjected to cumulative ACh concentrations (10^–9^–10^–4^ M) every 15 min. Isometric tension alterations were measured via an isometric tension transducer (MLT0420, AD Instruments, Australia) and recorded using AD Instruments PowerLab Chart software. Atropine (0.1 µM) was used as a positive control and added 15 min before ACh exposure.

### Bronchoalveolar lavage fluid (BALF) analysis

Bronchoalveolar lavage was performed three times, each time using 0.5 mL of warm PBS on the right lung. The combined BALF was centrifuged at 500 g for 10 min at 4°C. Cell-free supernatants were preserved at – 80°C for cytokine or chemokine analyses. The cell pellets were resuspended in 0.1 mL of PBS containing 1% FBS, and leukocyte differential counts were conducted using a BC-5000 Vet auto hematology analyzer (MINDRAY Medical International Co., Ltd., Shenzhen, China).

### Quantification of HDM-specific IgE

Cardiac puncture was used to collect blood samples, which were allowed to clot at room temperature for 30 min. Post-centrifugation at 1000 g for 10 min at 4ºC, sera were harvested for HDM-specific IgE quantification using an ELISA kit (BioLegend, Cat:432404) with modifications. Briefly, Nunc MaxiSorp ELISA Plates (BioLegend, Cat: 423501) were coated overnight at 4°C with mouse IgE capture antibodies, blocked, and then incubated with serum samples for 2 h. Biotinylated HDM extract (CITEQ, Cat: 02.01.88) was added for 1 h, followed by streptavidin-conjugated HRP. The reaction was developed with TMB substrate for 20 min, stopped with 2 M H_2_SO_4_, and absorbance at 450 nm was measured using a Synergy H1 microplate reader (BioTek). Optical density (OD) values were used to represent HDM-specific IgE levels.

### ELISA and multiplex immunoassay

Cytokines IL-4, IL-5, IL-6, and IL-13 in BALF were quantified using commercial sandwich ELISA kits from BioLegend (Cat: 431101, 431201, 431301) and Invitrogen (Cat: 88-7137-88), following the manufacturer’s guidelines. In addition, cytokine and chemokine in cell culture supernatants were analyzed using a multiplex immunoassay with a Luminex 200 system (Luminex, Austin, Texas, USA) and the Bio-Plex Pro Mouse Cytokine 23-plex kit (Bio-Rad, Cat: M60009RDPD), adhering to the manufacturer's recommendations. The lower limit of detection (LLOD) and upper limit of detection (ULOD) for both the ELISA and multiplex immunoassays are detailed in Supplementary Table 1.

### Histology and immunohistochemistry (IHC)

Mouse left lungs were fixed in 4% phosphate-buffered formaldehyde solution for 24 h or more. Tissues were embedded in paraffin, sectioned at 4 µm, and stained with hematoxylin and eosin (H&E) for inflammation assessment or periodic acid-Schiff (PAS) for mucus secretion evaluation under a Nikon 80I microscope. The inflammation severity was assessed by semi-quantitative scoring (0–4: 0, none; 1, few cells; 2, a ring of inflammatory cells with 1 cell layer deep; 3, a ring of inflammatory cells with 2–4 cells deep; 4, a ring of inflammatory cells with more than 4 cells deep). Numerical scores for the abundance of PAS-positive goblet cells in each airway were counted and expressed as a percentage of epithelial cells. Ten fields per section were scored blindly by two pathologists.

For IHC of CC10, sections were deparaffinized, rehydrated, and antigen-retrieved. Non-specific binding was blocked with 10% rabbit serum in PBS. Sections were incubated with anti-mouse CC10 antibodies (Abcam, Cat: ab40873) at 1:1000 dilution, followed by HRP-conjugated secondary antibody incubation. Visualization was done with diaminobenzidine (DAB) and hematoxylin counterstaining. Images were captured with a Nikon 80I microscope and analyzed using ImageJ software.

### Generation and culture of bone marrow-derived DCs (BMDCs)

Mice were euthanized under 5% isoflurane anesthesia and sterilized with 75% ethanol. Femurs and tibias were then excised, and the surrounding muscle and connective tissue were removed. Marrow cavities were flushed with ice-cold RPMI-1640 medium, using 25-gauge needle syringes. The isolated bone marrow cells underwent erythrocyte lysis with RBC lysis buffer (BioLegend, cat: 420,301), followed by resuspension in RPMI-1640 medium supplemented with 10% FBS, penicillin (100 U/mL), and streptomycin (100 mg/mL) at a density of 1 × 10^6^ cells/mL. The cells were cultured at 37°C in 5% CO_2_ with 20 ng/mL recombinant murine GM-CSF (R&D, cat: 415-ML) and 10 ng/mL recombinant murine IL-4 (R&D, cat: 404-ML). On day 3, non-adherent cells and 75% of the culture media were replaced with fresh media containing GM-CSF and IL-4. On day 5, half of the culture media was refreshed. On day 7, non-adherent cells were collected as immature BMDCs or further matured by replacing half of the media and adding 100 ng/mL LPS for an additional day.

### Isolation of lung DCs

Lungs were harvested, minced into 1-mm pieces, and digested for 45 min at 37 °C in digestion buffer (RPMI 1640 medium with 1 mg/mL collagenase IV and 200 U/mL DNase I), shaking at 250 rpm. Digested tissues were strained through a 70 μm sterile cell strainer. Erythrocytes were lysed, and lung cells were obtained via centrifugation. Lung DCs were isolated using CD11c-positive selection with UltraPure MicroBeads (Miltenyi Biotec, Cat: 130-125-835) and LS columns (Miltenyi Biotec, Cat: 130-125-835) as per the manufacturer's protocol. Typically, over 95% of the purified cells were CD11c^+^.

### T cell activation assay

T cells, isolated from wild-type C57BL/6 mouse splenocytes using a negative selection kit (Miltenyi Biotec, Cat: 130-095-130), were seeded into flat-bottomed 96-well plates pre-coated with 10 µg/mL anti-CD3 mAb (Clone:145-2C1) (BD Biosciences, Cat: 553,057) and cultured in RPMI 1640 medium with or without CC10 (1 µg/mL) for 48 h, then stained with APC-anti-CD28 for flow cytometric analysis. For some assays, T cells were co-stimulated with immobilized anti-CD3 mAb (10 µg/mL) and soluble anti-CD28 mAb (Clone: 37.51, 2 µg/mL) (BD Biosciences, Cat: 553294), and CD25 and CD69 expression levels were quantified by flow cytometry.

### Cellular uptake assay

Immature BMDCs were plated in 12-well plates and pre-treated with PBS, CC10 (1 μg/mL), or dexamethasone (10 μM) for 6 h. Cells were then incubated with1 mg/mL FITC-dextran (Sigma-Aldrich, Cat: 46,945) at 37 °C for 1 h, washed with ice-cold PBS, and analyzed for FITC-dextran uptake by flow cytometry. Uptake assessments in pulmonary DCs from wild type and *Cc10*^*−/−*^ mice were conducted at various time points over a period of 1–24 h.

### Mixed lymphocyte reaction assay

In the mixed lymphocyte reaction (MLR) assay, CD4^+^ T cells specific to OVA peptides were isolated from OT-II mouse spleens using the mouse CD4^+^ T Cell Isolation Kit (Miltenyi Biotec, Cat: 130-104-454) and subsequently stained with CFSE (0.5 μM) for proliferation tracking. BMDCs from wild-type C57BL/6 mice were cultured, then pulsed overnight with OVA_323-339_ peptides (Sigma Aldrich, Cat: O1641) at 2 μg/mL, with or without the addition of CC10 (1 μg/mL) or dexamethasone (10 μM). These BMDCs were co-cultured with CD4^+^ T cells from OT-II mice at a 1:10 ratio (DCs:T cells) in round-bottomed 96-well plates for 3 days. Following the co-culture, supernatants were harvested for cytokine quantification using the Multiplex Immunoassay (Millipore). The expression of CD25 and CD69 and the dilution of CFSE in CD4^+^ T cells, indicative of activation and proliferation, were assessed via flow cytometry. Additionally, to evaluate T cell differentiation, naïve CD4^+^ T cells from OT-II mice were co-cultured with OVA_323-339_ peptide-loaded allogeneic BMDCs in the presence of either CC10 or dexamethasone. The intracellular levels of IL-4 and IL-17A in these cells were subsequently analyzed through flow cytometry.

### Flow cytometric analysis

Cells subjected to specific treatments were blocked with anti-CD16/32 (93) (BD Biosciences, Cat: 101,302) in flow staining buffer (PBS with 2 mM EDTA and 0.5% FBS) for 10 min at 4 °C, followed by staining with fluorescently conjugated anti-mouse monoclonal antibodies at 4 °C in darkness for 30 min. Fixable Viability Dye eFluor 780 Stain (eBioscience, Cat: 65-0865-18) was utilized to exclude dead cells. Surface markers including CD11c-BUV395 (HL3), MHC II-FITC (2G9), CD45-BV510 (30-F11), CD11b-PerCP-Cy5.5 (M1/70), CD103-PE (2E7), CD86-BV421 (GL-1), CD40-APC (3/23), CCR7-AF647 (4B12), CD3-FITC (17A2), CD4-APC (RM4-5), CD25-PE-Cy7 (PC61), CD69-APC (H1.2F3) and CD28-APC (37.51) were assessed. Intracellular cytokines IL-4-PE-Cy7 (11B11) and IL-17A-PE (TC11-18H10) were stained using the Cytofix/Cytoperm Fixation/Permeabilization Kit (BD Biosciences, Cat: 554,714). Detail information for each FACS antibody used is provided in Supplementary Table 2. Post-staining, samples were washed, analyzed on an Attune NxT flow cytometer (ThermoFisher, MA, USA), and data were processed with FlowJo software (BD Biosciences).

### Quantitative reverse transcription polymerase chain reaction (RT-qPCR)

Total RNA was extracted from lung tissues or sorted lung DCs using Trizol reagent (Invitrogen, Cat: 15,596,026), followed by reverse transcription with the Revert Aid First Strand cDNA Synthesis Kit (Thermo Scientific, Cat: K1622). qPCR was conducted using SYBR green reagent (TOYOBO, Cat: QPK-201) on a LightCycler 96 System (Roche, Basel, Switzerland). Primer sequences are detailed in Supplementary Table 3. *Gapdh* served as the internal control gene, with relative gene expression calculated via the *ΔΔCt* method.

### Western blotting

BMDCs were pre-treated with CC10 (1 μg/mL) for 2 h, followed by stimulation with 100 ng/mL LPS. Cells were collected at designated time points (0–120 min), lysed in RIPA buffer with protease and phosphatase inhibitors, and proteins were resolved by 12% SDS-PAGE and transferred onto PVDF membranes (Millipore, Cat: IPVH00010). Blots were probed with antibodies against phospho-IκBα (Ser32) (Clone 14D4, Cell Signaling Technology, Cat: 2859), total IκBα (Clone L35A5, Cell Signaling Technology, Cat: 4814), phospho-p65 (Ser536) (Clone 93H1, Cell Signaling Technology, Cat: 3033), and total p65 (Clone D14E12, Cell Signaling Technology, Cat: 8242). Enhanced chemiluminescence was used for signal detection, imaged on an Amersham Imager 600 (GE Healthcare, USA). β-actin served as the loading control, and band intensities were quantified using ImageJ software.

### Statistical analysis

Data are presented as mean ± SEM. Group differences were assessed using One-way ANOVA followed by either LSD or Games-Howell tests, depending on the data distribution. For non-parametric datasets, the Kruskal–Wallis test was employed, with post-hoc comparisons conducted using Dunnett’s test. AHR experiments and tracheal ring contraction measurements were analyzed using Two-way ANOVA. Two-tailed Pearson correlation was utilized for association analyses. Statistical significance was established at a p-value less than 0.05.

## Results

### Decreased CC10 expression in the serum of asthmatic patients and the lungs of HDM-induced asthma models

**Fig. 1:**
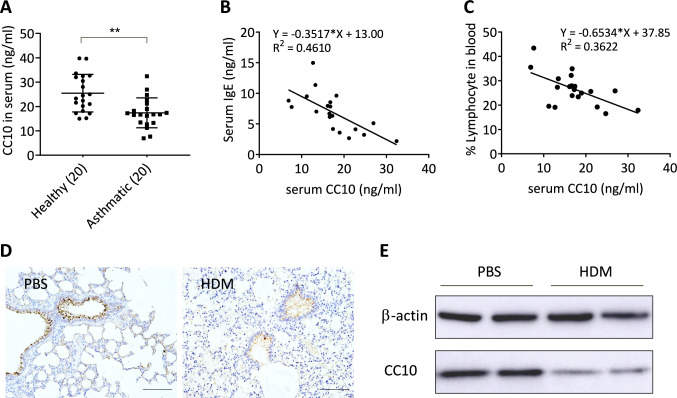
Asthma-associated reduction in CC10 expression in human and murine subjects. **A** ELISA quantification of CC10 in peripheral blood from healthy (n = 20) and asthmatic (n = 20) individuals. **P < 0.01 via unpaired 2-tailed Student *t*-test. **B**, **C** Inverse correlation of serum CC10 with IgE level (**B**) and lymphocyte percentage (**C**) in asthma (n = 20). **D** Immunohistochemistry of lung sections from control and HDM-exposed mice. Scale bar = 500 μm. **E** Western blot analysis of CC10 in lung tissues from control and HDM-exposed mice. Two independent experiments were performed

In our study, we focused on CC10, a key immunoregulatory and anti-inflammatory protein secreted predominantly by Clara cells. Initially, we quantified CC10 levels in the serum of asthmatic patients using an ELISA assay. Figure 1A demonstrates a notable reduction in CC10 expression in asthmatic patients compared to healthy individuals. Intriguingly, serum CC10 levels were inversely associated with both IgE concentration (r =  −0.68, *P* < 0.05; Fig. 1B) and the proportion of peripheral blood lymphocytes (r = -−0.60, *P* < 0.05; Fig. 1C) in these patients.

Additionally, we investigated the impact of HDM sensitization and challenge on CC10 expression in a murine allergic asthma model. Figure 1D and E reveal that while CC10 is primarily present in the airway epithelium, its levels in the lungs of HDM-challenged mice were significantly lower than those in the PBS-treated control group. These findings suggest that CC10 may play a crucial role in modulating allergic inflammatory responses in asthma, highlighting its potential as a biomarker or therapeutic target in this condition.

### Augmented HDM-induced allergic airway inflammation in CC10 deficient mice

**Fig. 2:**
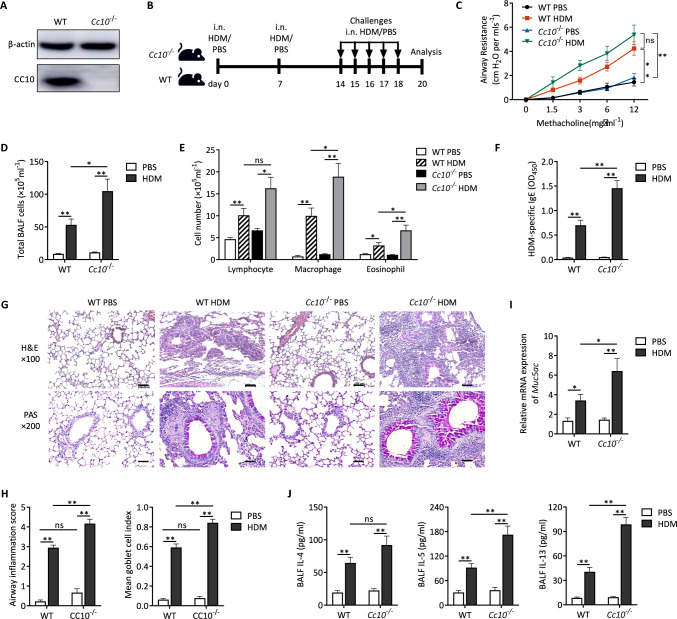
Enhanced allergic asthma in *Cc10*^*−/−*^ mice post HDM exposure. **A** Western blot showing absence of CC10 in *Cc10*^*−/−*^ lung tissue. **B** HDM sensitization/challenge protocol for wild type and *Cc10*^*−/−*^ mice, with PBS controls. Analysis occurred 2 days post-final challenge. **C** Airway resistance to MCh increments; normalized to baseline (n = 6/group). **D**, **E** BALF cell counts (n = 6/group). **F** ELISA quantification of HDM-specific IgE (n = 6/group). **G** Lung histology stained with H&E or PAS; scale bar = 200 μm. **H** Quantitative assessment of inflammatory infiltration and goblet cell hyperplasia (n = 6/group). **I** Quantification analysis for *Muc5ac* mRNA expression in mice lung tissues (n = 4/group). **J** ELISA determination of IL-4, IL-5, and IL-13 in BALF (n = 6/group). Data are mean ± SEM. *P* values: one-way ANOVA with Games-Howell test (**D**–**I**), two-way ANOVA (**C**). *P < 0.05, **P < 0.01, *ns* not significant

To ascertain the protective role of CC10 against allergic airway inflammation, we engineered CC10 gene knockout (*Cc10*^*−/−*^) mice, effectively eliminating CC10 protein from lung tissue (Fig. 2A). Both *Cc10*^*−/−*^ and wild-type (WT) mice were subjected to intranasal sensitization and challenge with HDM extract, as depicted in Fig. 2B.

Initially, we observed an increase in HDM-induced AHR in *Cc10*^*−/−*^ mice compared to WT mice, although this difference was not statistically significant (Fig. 2C). Notably, the total inflammatory cell count in BALF of HDM-treated *Cc10*^*−/−*^ and WT mice was significantly higher than that in vehicle-treated control mice, reflecting increases in lymphocytes, alveolar macrophages, and eosinophils. These increases were more pronounced in *Cc10*^*−/−*^ mice (Fig. 2D and E). Furthermore, HDM-treated *Cc10*^*−/−*^ mice exhibited a significant upsurge in serum HDM-specific IgE levels compared to HDM-treated WT mice (Fig. 2F). Histological analysis revealed increased bronchial wall thickening, peribronchiolar cell infiltration, and a higher number of PAS-positive cells in HDM-treated *Cc10*^*−/−*^ mice (Fig. 2G and H). Additionally, mRNA levels of *Muc5ac* were higher in HDM-treated *Cc10*^*−/−*^ mice than in HDM-treated WT mice (Fig. 2I). Correspondingly, levels of IL-4, IL-5, and IL-13 in BALF were elevated in HDM-treated *Cc10*^*−/−*^ mice compared to their WT counterparts (Fig. 2J).

In summary, in response to HDM exposure, *Cc10*^*−/−*^ mice exhibited heightened Th2 immune responses, intensified eosinophilic airway inflammation, increased mucous cell hyperplasia, and augmented AHR. These results collectively underscore the significant protective role of CC10 in countering HDM-induced allergic airway inflammation.

### Recombinant CC10 protein attenuates HDM-induced allergic airway inflammation

**Fig. 3:**
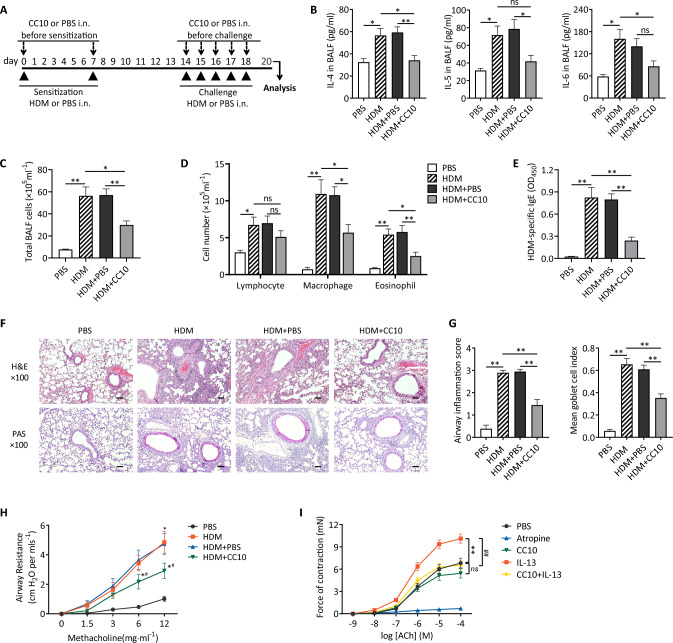
Alleviation of allergic airway inflammation via CC10 treatment. **A** Experimental outline. Mice underwent HDM or PBS exposure, with or without preceding intranasal CC10 (25 μg) administration. **B** ELISA analysis of IL-4, IL-5, and IL-6 in BALF (n = 6/group). **C**, **D** BALF cell counts (n = 6/group). **E** HDM-specific IgE quantification via ELISA (n = 6/group). **F** Lung sections stained with H&E or PAS; scale bar = 200 μm. **G** Quantitative analysis of inflammation and goblet cell hyperplasia (n = 6/group). **H** Airway resistance to Mch, normalized to baseline (n = 6/group). **I** Tracheal ring contraction response to ACh post CC10/IL-13 treatment (n = 5/group). Data are mean ± SEM. *P* values: one-way ANOVA with Tukey–Kramer test (**B**–**F**), two-way ANOVA (G, H). *P < 0.05, **P < 0.01, *ns* not significant

After observing that CC10 deficiency exacerbates antigen-induced allergic airway inflammation, we explored the therapeutic potential of exogenous CC10 protein in mitigating inflammation in HDM-induced asthma models. Mice were sensitized through two HDM airway instillations and subjected to repeated intranasal HDM challenges. Recombinant murine CC10 protein was administered intranasally 2 h prior to each antigen exposure (Fig. 3A). The optimal CC10 dosage, determined as 25 μg/mice, was identified by evaluating changes in AHR, leukocyte counts in BALF, antigen-specific IgE levels in serum, and IL-4 and IL-5 levels in BALF post-administration (Fig. S1).

To assess the Th2-mediated response to HDM, we measured IL-4, IL-5, and IL-6 levels in BALF using ELISA. CC10-treated mice exhibited significantly lower Th2 cytokine levels compared to both HDM-challenged and vehicle-treated groups (Fig. 3B). In addition, BALF from HDM-challenged mice contained increased inflammatory cells such as eosinophils, alveolar macrophages, and lymphocytes, confirming the role of HDM in provoking allergic airway inflammation. Treatment with CC10 resulted in a significant reduction in the total number of inflammatory cells in the BALF. Specifically, there was a marked decrease in eosinophils and alveolar macrophages (Fig. 3C and D). Furthermore, there was a marked decrease in serum levels of HDM-specific IgE following CC10 administration (Fig. 3E). Lung histopathology revealed that CC10 treatment significantly inhibited peribronchial and perivascular cellular infiltration, goblet cell hyperplasia, and mucus production compared to vehicle treatment (Fig. 3F and G). These findings collectively highlight the potent anti-inflammatory effects of CC10 in the context of allergic airway inflammation.

Given the pivotal role of airway inflammation in driving AHR in allergic asthma, we further investigated the effect of CC10 on AHR in response to HDM. CC10 treatment resulted in a significant reduction of HDM-induced AHR at both 6 mg/mL and 12 mg/mL doses of aerosol MCh compared to controls (Fig. 3H). Recognizing IL-13, produced by Th2 cells in the lung, as a critical regulator of AHR[24], tracheal rings from untreated mice were incubated with IL-13 for one hour before evaluating their contractile response to ACh. Tracheal rings exposed to IL-13 exhibited enhanced contractile responses to ACh, a reaction significantly attenuated by CC10 pre-treatment. Notably, CC10 did not influence the contraction of tracheal rings induced by ACh alone (Fig. 3I), underscoring specific role of CC10 in dampening Th2 cytokine-induced AHR enhancement. Collectively, these findings suggest that CC10 attenuates allergic airway inflammation primarily by inhibiting Th2-driven immune responses.

### CC10 has no direct inhibition on T cell activation

**Fig. 4:**
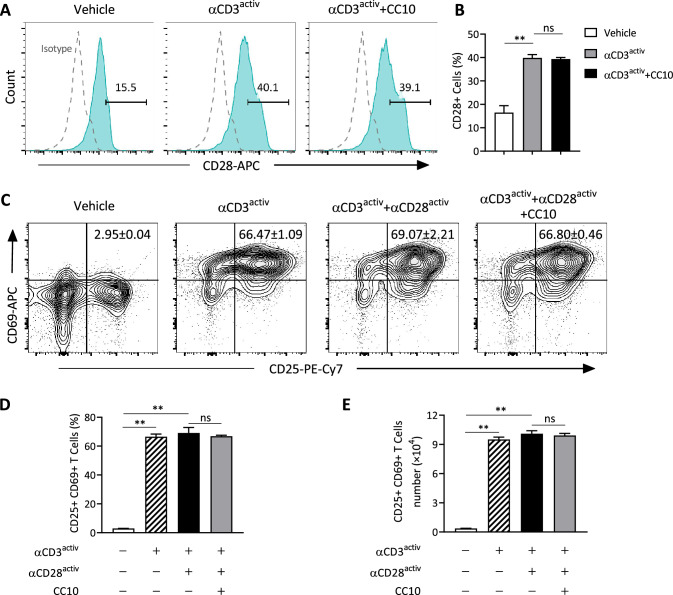
CC10 did not directly affect T cell activity. **A**, **B** Naïve mouse CD3^+^ T cells, activated with anti-CD3 mAb, were treated with CC10 (1 μg/mL) for 48 h. Flow cytometry was used to assess CD28 expression (A) and quantify CD28^+^ cells (B). **C** T cells, stimulated with immobilized anti-CD3 mAb alone or in combination with soluble anti-CD28 mAb, and CC10 (1 μg/mL) for 48 h, were analyzed for CD25 and CD69 levels via flow cytometry. **D**, **E** Quantitative analysis of CD25^+^CD69^+^ T cells was conducted. Representing three experiments, data are mean ± SEM. *P* values derived from one-way ANOVA with Tukey–Kramer test. *P < 0.05, **P < 0.01, *ns* not significant

To elucidate the mechanisms underlying the anti-inflammation function of CC10, we initially investigated its direct inhibitory effect on T lymphocyte activation in vitro. CD28, a proinflammatory costimulatory receptor on T cells, typically up-regulates following TCR-induced stimulation. As anticipated, CD28 surface expression on T cells augmented upon anti-CD3 mAb stimulation, part of the TCR complex. Nonetheless, CC10 treatment did not impair the elevated CD28 levels on anti-CD3 activated T cells (Fig. 4A and B). Further, we explored the effect of CC10 on activation markers CD69 and CD25 on T cells. Despite significant up-regulation of CD25 and CD69 post 24-h activation with immobilized anti-CD3 mAb alone or combined with soluble anti-CD28, the frequencies and total counts of CD25^+^CD69^+^ T cells remained unchanged between the anti-CD3/CD28 stimulated and CC10-treated groups, indicating that CC10 has no direct inhibitory action on T cell activation (Fig. 4C–E).

### CC10 modulates the phenotype and function of lung DCs in asthmatic mice

**Fig. 5:**
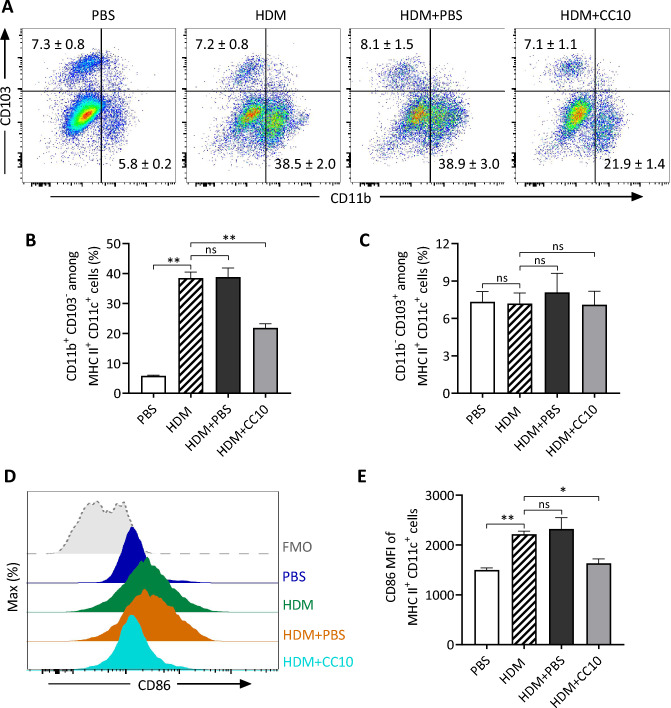
CC10 reduces CD11b^+^CD103^−^ subsets and CD86 expression in lung DCs of HDM-induced Asthma. **A** Lung single-cell suspensions analyzed for DC subsets via flow cytometry, with gating shown in Fig E2. **B**, **C** Percentages of CD11b^+^CD103^−^ (B) and CD11b^-^CD103^+^ (C) lung DCs were quantified (n = 6/group). **D**, **E** Flow cytometric analysis and mean fluorescence intensity of CD86 on lung DCs (n = 6/group). Data are mean ± SEM. *P* values calculated via one-way ANOVA with Games-Howell test. *P < 0.05, **P < 0.01, *ns* not significant

Given that CC10 does not directly inhibit T lymphocyte activation in vitro, we hypothesized its potential to modulate T cell-mediated adaptive immune responses via altering DC functions. We first examined the impact of CC10 on the phenotype and function of lung DCs in HDM-induced allergic asthma models. Flow cytometry analysis of murine single cell suspensions indicated a significant increase in the CD11b^+^CD103^-^ subset (cDC2) in lung DCs from HDM-primed mice, while CD11b^-^CD103^+^ (cDC1) levels remained unchanged. Intranasal CC10 administration significantly reduced cDC2 numbers in the lungs of HDM-induced asthmatic mice compared to PBS-treated controls (Fig. S2A and B, Fig. 5A–C). Moreover, HDM challenge prompted lung DCs to mature and activate, evidenced by increased CD86 expression, which was notably diminished by CC10 treatment (Fig. 5D and E)

**Fig. 6:**
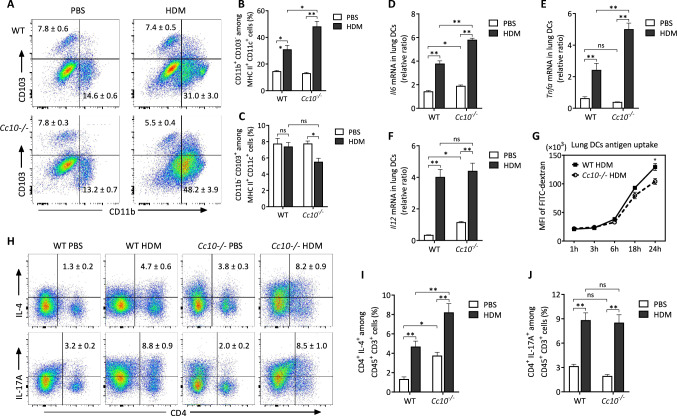
Augmented DCs activation and Th2 expansion in *Cc10*^*−/−*^ mice post-HDM challenge. **A** Flow cytometry of lung CD11b^+^CD103^−^ and CD11b^−^CD103^+^ DCs (gated on live CD45^+^CD11c^+^HLA-DR^+^ cells). **B**, **C** Quantification of DC subsets (n = 6/group). **D**–**F** qRT-PCR analysis of *Il6, Tnfa, Il12* mRNA levels in lung DCs post-HDM exposure (n = 3/group). **G** FITC-dextran uptake by lung DCs measured via flow cytometry. Data are representative for three independent experiments (n = 4/group). **H** Flow cytometry of Th2 (CD4^+^IL-4^+^) and Th17 (CD4^+^IL-17A^+^) cells in lungs. **I**, **J** Quantification of Th cell subsets (n = 6/group). Data are mean ± SEM, representing three experiments. *P* values from Student’s* t*-test (**G**) and one-way ANOVA with Games-Howell test. *P < 0.05, **P < 0.01, *ns* not significant

Next, we compared lung DC phenotypes and functions in WT and *Cc10*^*-/-*^ mice under HDM-induced allergic airway inflammation. Flow cytometry revealed a significant increase in cDC2 cells in the lungs of HDM-challenged *Cc10*^*-/-*^ mice compared to WT mice, while cDC1 cells remained unchanged (Fig. 6A–C). CC10 deletion led to a marked reduction in cDC1 cells in the lungs of *Cc10*^*-/-*^ mice exposed to HDM, an alteration not observed in WT mice. qPCR assay indicated significant elevations in *Il6*, *Tnfa*, and *Il12* mRNA levels in lung DCs of both *Cc10*^*-/-*^ and WT mice following HDM exposure, with *Cc10*^*-/-*^ asthmatic mice showing significantly higher *Il6* and *Tnfa* mRNA levels than their WT counterparts (Fig. 6D–F). Additionally, lung DCs from *Cc10*^*-/-*^ mice demonstrated a reduced capacity for Dextran antigen uptake and endocytosis *ex vivo* compared to WT, indicating enhanced maturation and activation in *Cc10*^*-/-*^ lung DCs (Fig. 6G). Moreover, the proportion of IL-4^+^CD4^+^ Th2 cells and IL-17A^+^CD4^+^ Th17 cells in the lung (gating strategy was shown in Fig. S2C and D) significantly increased after HDM sensitization and challenge in both WT and *Cc10*^*-/-*^ mice, with *Cc10*^*-/-*^ mice exhibiting higher levels of Th2 cells, but not Th17 cells, compared to their WT counterparts (Fig. 6H–J).

### CC10 suppresses immune activation in BMDCs

**Fig. 7:**
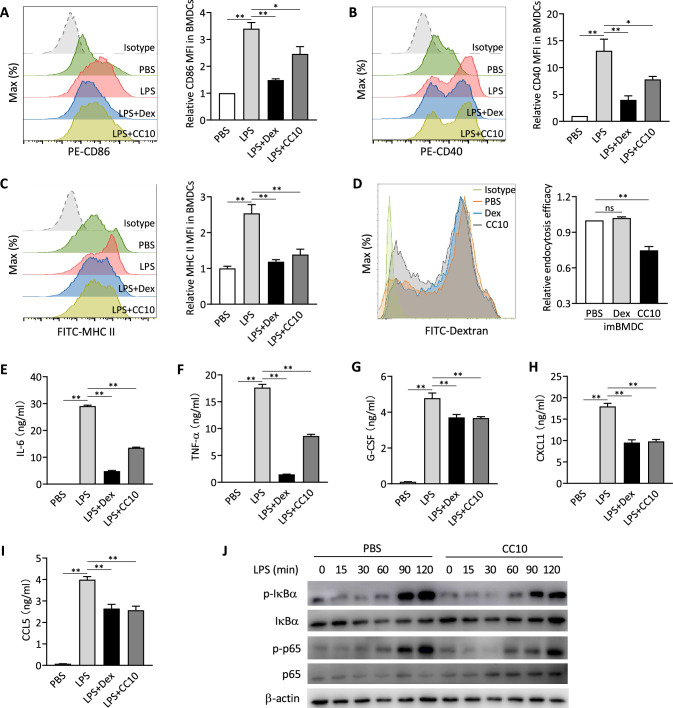
CC10 modulates immunological activity of BMDCs In Vitro. **A**–**C** Flow cytometric analysis of CD86, CD40, HLA-DR on BMDCs post CC10 or dexamethasone (Dex) and LPS (200 ng/mL) treatment. The graph shows averages from 4 independent experiments. **D** FITC-dextran endocytosis by immature BMDCs. Data from 3 independent experiments. **E**–**I** ELISA for cytokines in supernatants of LPS-stimulated BMDCs pre-treated with CC10 or Dex (n = 3/group). **J** Western blot analysis of IκBα and p65 phosphorylation in LPS-stimulated BMDCs with or without CC10. β-actin used as loading control. Data from two experiments with similar results. All data are shown as mean ± SEM. *P* values from one-way ANOVA with Games-Howell test. *P < 0.05, **P < 0.01, *ns* not significant

We further investigated whether CC10 can directly act on DCs to regulate their functions by using cultured BMDCs. LPS-treated BMDCs exhibited increased surface expression of MHC II and costimulatory molecules CD86 and CD40, pivotal for T cell activation in adaptive immunity (Fig. 7A–C). Furthermore, LPS stimulation significantly elevated the secretion of pro-inflammatory cytokines, including IL-6, TNFα, and G-CSF, alongside chemokines like CXCL1 and RANTES (Fig. 7E–I). Dexamethasone demonstrated potent anti-inflammatory effects by suppressing both the expression and production of these markers.

Pre-treatment of BMDCs with CC10 effectively suppressed LPS-induced pro-inflammatory responses, indicated by the marked down-regulation of MHC II, CD86, and CD40 surface expression (Fig. 7A–C), while not significantly affecting CD80 (data not shown). CC10 similarly reduced CD86 expression in LPS-treated JAWS II cells, an immortalized immature DC line (Fig. S3A). Moreover, CC10 significantly decreased the uptake and endocytosis of FITC-dextran by immature BMDCs (Fig. 7D) and JAWS II cells (Fig. S3B), an effect not observed with dexamethasone. CC10 also notably reduced the release of pro-inflammatory cytokines and chemokines compared to the LPS-stimulated group, with its inhibitory effects on G-CSF, CXCL1, and RANTES production mirroring those of dexamethasone (Fig. 7E–I). Importantly, these effects were specific to CC10's regulatory action, as evidenced by the maintained viability of CC10-treated cells (Fig. S4).

To elucidate the molecular mechanisms underlying the inhibitory effects of CC10 on LPS-activated BMDCs, we assessed the TLR/NF-κB signaling pathways. BMDCs pre-treated with CC10 showed significantly lower levels of phosphorylated IκBα and phosphorylated NF-κB subunit p65 after LPS stimulation compared to PBS-treated controls (Fig. 7J), indicating that CC10 mitigates LPS-induced inflammation in BMDCs by curbing NF-κB signaling activation.

### CC10 suppresses antigen-specific CD4^+^ T cell response via DCs

**Fig. 8:**
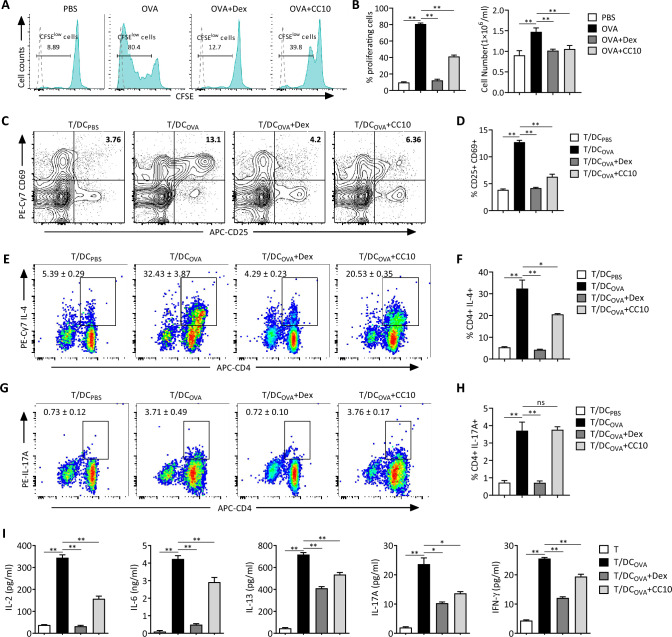
CC10 inhibits antigen-specific CD4 + T cell response via DCs. OVA-specific CD4^+^ T cells from OT-II mice were co-cultured with BMDCs at a ratio of 1:10 (BMDCs/T cells), with/without CC10 (1 μg/mL) or Dex (10 μM), and OVA323-339 peptide. **A** Flow cytometry for CFSE dilution in CD4^+^ T cells. Unlabeled cells (blue-dashed histogram) are shown for comparison. **B** Quantification of proliferating cells.** C** Flow cytometry for CD25 and CD69 expression on CD4^+^ T cells. **D** Quantification of CD25^+^CD69^+^ cells.** E** Flow cytometry for IL-4-producing CD4^+^ T cells in the culture. **F** Quantification of IL-4^+^CD4^+^ cells. **G** Flow cytometry for IL-17A-producing CD4^+^ T cells in the culture. **H** Quantification of IL-17A^+^CD4^+^ cells.** I** ELISA for cytokines in culture supernatants (n = 4/group). Data of (**B**, **D**, **F**, **H**) shows averages from three independent experiments, and all data are presented as mean ± SEM. *P* values from one-way ANOVA with Games-Howell test. *P < 0.05, **P < 0.01, *ns* not significant

Although CC10 has no direct suppressive effects on T cell activation, it can act directly on DCs and regulate their phenotypes and functions, prompting the question of whether CC10 inhibits CD4^+^ T cell immune activation through DCs. To this end, we used the MLR assay, in which OVA-specific CD4^+^ T cells isolated from OT-II mice were co-cultured with OVA-loaded BMDCs at a ratio of 1:10 (DCs/T-cells), and examined the effect of CC10 on the proliferation, activation, and differentiation of CD4^+^ T cells. CFSE dilution analysis showed that dexamethasone or CC10 treatment significantly reduced the proliferative response of OVA-specific CD4^+^ T cells induced by antigen-loaded BMDCs, as evidenced by the decreased percentage and number of CFSE-diluted CD4^+^ T cells (Fig. 8A and B). We measured the activation of OVA-specific CD4^+^ T cells in MLR cultures by assessing CD25 and CD69 expression via flow cytometry. Flow cytometry analysis demonstrated that both dexamethasone and CC10 effectively suppressed the proportion of CD25^+^CD69^+^CD4^+^ T cells following stimulation with OVA_323-339_ peptide-loaded BMDCs (Fig. 8C and D).

Additionally, we observed that culturing with OVA-loaded BMDCs markedly enhanced the differentiation of IL-4^+^CD4^+^ Th2 and IL-17A^+^CD4^+^ cells from naïve Th cells. However, CC10 significantly diminished the Th2 cell population levels, without affecting the Th17 cell population in MLR cultures (Fig. 8E–H). OVA-specific CD4^+^ T cells exhibited increased cytokine production (IL-2, IL-6, IL-13, IL-17A, and IFN-γ) following stimulation with OVA_323-339_ peptide-loaded BMDCs. Both CC10 and dexamethasone treatments reduced these cytokine secretions in MLR cultures, though the inhibitory effect of CC10 was less pronounced than dexamethasone (Fig. 8I). Moreover, CC10 notably decreased the expression levels of TNF-α and IL-12 in the MLR supernatant, without affecting IL-10 secretion (Fig. S5A-C).

## Discussion

CC10, known for its protective role in pulmonary inflammatory disorders, has its mechanistic action still under investigation. In our study, we assessed CC10 levels in the serum of asthmatic patients and the lung tissues from HDM-induced murine asthma models. Consistent with previous findings [23, 25], we observed a significant reduction in CC10 expression in both contexts, highlighting its potential role as a biomarker for allergic airway inflammation. Analyzing asthmatic patients, we discovered significant inverse correlations between serum CC10 concentrations and both serum IgE levels and peripheral blood T cell counts. This aligns with a recent study showing a similar inverse relationship between plasma CC10 levels and eosinophilic indicators in both blood and sputum of asthmatic patients [26]. Collectively, these observations, alongside the documented anti-inflammatory properties of CC10, bolster the hypothesis that a reduction in CC10 levels may contribute to the pathogenesis and severity of asthma by diminishing its capacity to regulate inflammatory cytokines. This not only reinforces the protective role of CC10 against pulmonary inflammation but also highlights its potential as a key regulatory factor within the complex immunological landscape of asthma.

To investigate the direct impact of CC10 on the development of allergic airway inflammation characteristic of asthma, we analyzed the changes in airway inflammation within HDM-primed *Cc10*^*−/−*^ mice. As anticipated, these mice demonstrated significantly intensified allergic Th2 inflammatory responses compared to their WT counterparts. This was evidenced by elevated inflammatory cell counts, increased levels of Th2 cytokines such as IL-4, IL-5, and IL-13 in BALF, augmented HDM-specific serum IgE levels, pronounced peribronchial inflammatory cell infiltration, goblet cell hyperplasia, and enhanced mucus hypersecretion. Furthermore, the *Cc10*^*−/−*^ mice exhibited more pronounced MCh-induced AHR following HDM exposure than the WT mice. These observations align with previous studies [27, 28] indicating that CC10 deficiency leads to exacerbated pulmonary eosinophilic inflammation in mouse models of asthma induced by OVA. Consequently, our findings suggest a broader protective role for CC10 in safeguarding the host against antigen-induced allergic airway inflammation and the associated Th2 lymphocyte reactivity.

Given the role of diminished CC10 secretion in exacerbating lung inflammation, enhancing CC10 levels may provide a strategic approach for preventing and managing pulmonary inflammatory conditions. This hypothesis is supported by previous studies indicating the efficacy of recombinant CC10 protein treatment in ameliorating lung function and moderating inflammatory responses in various animal models, including COPD [29], acute respiratory distress syndrome [30], and allergic rhinitis [31]. Our current research further substantiates this notion, demonstrating that the administration of recombinant mouse CC10 protein to HDM-induced asthmatic mice notably reduced inflammatory cell counts, Th2 cytokine levels (IL-4, IL-5, and IL-6) in BALF, and serum HDM-specific IgE concentrations. Additionally, CC10 administration effectively diminished peribronchial inflammatory cell recruitment and infiltration, curtailed goblet cell hyperplasia, mucus secretion, and mitigated MCh-induced AHR. These observations are consistent with a previous study showing that CC10 overexpression via mucosal gene transfection curbed Th2 cytokine production and eosinophil infiltration in the lungs of CC10-deficient mice [32].

Interestingly, while exogenous CC10 did not influence the ACh-induced contractile response of tracheal rings directly, it notably subdued the IL-13-prompted enhancement of tracheal ring contractility. Given the critical role of IL-13 in orchestrating AHR in asthma, as demonstrated by Chiba et al. through the IL-13-mediated upregulation of RhoA protein via STAT6 activation [33], our findings suggest a potential mechanistic pathway wherein CC10 impedes IL-13 production or disrupts the IL-13/STAT6 signaling cascade, thereby attenuating AHR in asthma. These insights underscore the multifaceted role of CC10, not only as a mediator in pulmonary inflammation but also as a potential therapeutic agent in the modulation of asthma pathophysiology.

Our study, involving both *Cc10*^*−/−*^ mice and recombinant CC10 administration, confirms the significant role of CC10 in attenuating Th2-mediated inflammatory responses against allergens in allergic asthma. These findings raise an important question regarding the direct inhibitory effect of CC10 on T-cell responses. Our data indicate that CC10 administration did not alter the enhanced expression of CD28 on anti-CD3-activated T cells, nor did it affect the upregulation of activation markers CD69 and CD25 on T cells subjected to concurrent anti-CD3 and anti-CD28 stimulation. Hence, it appears that the suppression of Th2 response by CC10 in allergic inflammation is likely not exerted through direct interaction with T cells. This inference is consistent with earlier studies, which showed CC10 failed to suppress Th2 polarization of CD4^+^ T cells under specific polarizing conditions and to curb the production of Th2 cytokines by anti-CD3/CD28-stimulated naïve or differentiated CD4^+^ T cells [32, 34]. Moreover, while a decrease in OVA-induced T-cell proliferation was noted in CC10-treated splenocytes from OVA-sensitized mice, CC10 did not exhibit a consistent inhibitory effect on polarized Th2 cells stimulated with combined anti-CD3/anti-CD28 mAbs [32]. Additionally, CC10 did not directly affect the cytokine-driven differentiation of Th17 cells from naïve CD4^+^ T cells in vitro [31]. Taken together, these observations suggest that the regulatory effect of CC10 on antigen-induced T-cell responses likely involves interactions with antigen-presenting cells, such as DCs and macrophages, rather than exerting a direct action on T cells themselves. This revelation adds a new dimension to our understanding of the immunomodulatory role of CC10 in allergic asthma and underscores the complexity of its interaction with the cellular components of immune system.

DCs are recognized as the principal antigen-presenting cells, distinguished by their unique ability to activate naive T cells and initiate primary immune responses. They function as crucial mediators bridging innate and adaptive immunity in pulmonary inflammation [20, 35]. Specifically, within the conducting airways, DCs positioned beneath and within the epithelium shape T cell responses, notably through crosstalk with airway epithelial cells [36]. CC10, a prominent product of Clara cells in the airway epithelium, is posited to directly interact with DCs, potentially reshaping their immunological response patterns. In our quest to elucidate the immunomodulatory effect of CC10 on lung DCs in asthma, we examined the alterations in lung DC populations in a murine asthma model subsequent to CC10 administration. DCs are broadly classified into plasmacytoid dendritic cells (pDCs) and myeloid or conventional dendritic cells (cDCs). In the murine lung, cDCs comprise two primary subsets, discerned by their reciprocal expression of CD11b and CD103 integrins. Notably, cDC2, characterized by elevated CD11b and diminished CD103 expression, are adept at eliciting robust proinflammatory chemokine production [37], fostering Th2 differentiation [38], and facilitating Th2-mediated inflammatory cascades [21]. Our investigation observed a significant reduction in the CD11b^+^CD103^−^ cDC2 subset in the lungs following CC10 administration, whereas the CD11b^−^CD103^+^ cDC1 subset numbers remained constant. Conversely, *Cc10*^*−/−*^ mice manifested a pronounced upsurge in the cDC2 subset following HDM exposure compared to their wild-type counterparts. These findings lead us to propose that the CC10-mediated downregulation of the cDC2 subset significantly curtails the Th2-dominant inflammatory reaction to allergens, elucidating a pivotal pathway through which CC10 modulates asthma pathophysiology.

Upon capturing antigens, airway DCs migrate to the lung lymph nodes, where they undergo changes in cell morphology towards an activated mature phenotype. This is characterized by an augmented expression of MHC II molecules, facilitating the presentation of antigens to T cells, along with an upregulation in the expression of co-stimulatory molecules vital for T cell activation. CD86 is one of the key costimulatory molecules on DC surface and is highly expressed upon DC activation, serving as a ligand for CD28 or CTL-4 on T cells, thus triggering T cell activation. The critical role of CD86 in favoring the development of Th2 cells and driving Th2 immune responses to inhaled allergens has been well-documented [39–41]. In this study, we report a significant reduction in CD86 expression on lung DCs following CC10 treatment, with CC10 also substantially lowered CD86 expression on activated BMDCs stimulated with LPS. Moreover, CC10 notably suppressed the expression of both MHC II and CD40 on LPS-challenged BMDCs. Therefore, we postulate that CC10 suppresses lung DC maturation and activation by curtailing the expression of MHC II, and co-stimulatory molecules CD86 and CD40. This mechanism potentially delineates another pathway through which CC10 attenuates Th2-dominant allergic inflammatory reactions in asthmatic mice.

DCs have the capacity to release various cytokines, thereby modulating the immune activity of Th cells. Previous studies have indicated that within the lungs of mice with allergic asthma, DCs can activate the JAK/STAT3 signaling pathway by secreting IL-6, thereby promoting the differentiation and activation of Th2 and Th17 cells [42, 43]. TNF-α from DCs appears to potentiate activation of airway epithelial cells, thus amplifying the allergen-induced immune response in airways [44]. Our investigation into the *Cc10*^*−/−*^ asthma mouse model revealed that lung DCs exhibited substantially higher mRNA expressions of IL-6 and TNF-α compared to wild-type counterparts, 24 h after allergen exposure. Further, CC10 pretreatment notably inhibited the LPS-stimulated IL-6 and TNF-α production in BMDCs. Subsequent exploration demonstrated a significant increase in Th2 cell population in the lungs of *Cc10*^*−/−*^ mouse asthma model, indicating that CC10 might regulate Th2 cell differentiation and immune responses by suppressing IL-6 and TNF-α secretion from lung DCs. However, no significant change was noted in the Th17 cell population between *Cc10*^*−/−*^ and wild-type asthmatic mice. This discrepancy could be attributed to the dependence of mouse Th17 cell differentiation on TGF-β [45], and our study found that the regulatory effect of CC10 on TGF-β was negligible (data not shown). Interestingly, our findings are in line with previous studies that have shown an increase in the expression of CC10 in the airways after systemic allergen-specific immunotherapy for airway allergic inflammation. This increase in CC10 is paired with a significant decrease in the local Th2 response, despite no significant change in the Th17 cell population during the treatment [17, 46]. Thus, while CC10 appears to play a significant role in modulating Th2 cell-mediated immune responses through cytokine regulation in DCs, its effect on Th17 cell responses in the context of asthma remains limited.

We also noted that, following allergen stimulation, the IL-12 mRNA levels in lung DCs from *Cc10*^*−/−*^ asthmatic mice were comparable to those in wild-type counterparts. This finding may be attributed to the failure of CC10 to alter the number of the lung cDC1 subset in asthmatic mice. This explanation is supported by evidence indicating that lung cDC1 can release substantial amounts of IL-12, which helps inhibit the activation of Th2 cells and mitigate the exacerbation of allergic airway inflammation caused by chronic allergen exposure [47]. In addition, CC10 significantly reduced the levels of G-CSF produced by BMDCs upon LPS stimulation, concurrently diminishing the levels of chemokines CXCL1 and CCL5. G-CSF is renowned for fostering neutrophil development in the bone marrow and is implicated in increased neutrophil presence in airways, a hallmark of neutrophilic airway inflammation [48]. CXCL1, a member of the CXC chemokine subfamily, serves as a potent chemoattractant, particularly for neutrophils, guiding them to inflammation sites and contributing to neutrophilic inflammation [49]. CCL5, also known as RANTES, is adept at recruiting T lymphocytes and eosinophils in the peripheral immune system. Its elevated levels in the sputum of asthmatic patients have been associated with eosinophil recruitment in the airways [50]. Collectively, CC10 appears to modulate the secretion of cytokines and chemokines by lung DCs, thereby inhibiting the aggregation and activation of key immune cells like T lymphocytes, neutrophils, and eosinophils in the airways. This notion is reinforced by the observed decrease in these immune cells in the BALF of asthma mouse model treated with CC10, elucidating a potential pathway through which CC10 exerts its protective effects in allergic airway inflammation.

DCs are a highly heterogeneous cell population, with immature DCs or tolerogenic DCs exhibiting robust antigen uptake and processing abilities but limited antigen presentation capacity, leading to T cell anergy or immune tolerance [51]. Our study demonstrated that lung DCs from *Cc10*^*−/−*^ asthmatic mice displayed significantly lower uptake of Dextran compared to their wild-type counterparts. This implies that CC10 may facilitate the maintenance of an immature or immune-tolerant state in lung DCs, consequently dampening the inflammatory response to allergens in the lungs. Moreover, our in vitro observations revealed a marked decrease in antigen uptake by immature BMDCs upon treatment with CC10, without a corresponding shift to a mature phenotype. This underscores the multifaceted and complex regulatory effects of CC10 on the immune functions of DCs across different states, which requires to be further explored.

DCs engage pathogens through Toll-like receptors (TLRs), triggering a cascade of signaling pathways that culminate in their maturation and activation [52]. Central to this process is the transcription factor NF-κB (nuclear factor kappa B), a key player in TLR signaling and various immune responses [53]. Our study reveals that LPS exposure activated NF-κB signaling in BMDCs, marked by increased phosphorylation of IκB and p65 subunits. Intriguingly, CC10 pre-treatment notably reduced these phosphorylation levels in LPS-stimulated BMDCs. This implies that CC10 may downregulate TLR-mediated NF-κB activation, thereby inhibiting pathogen-induced DC activation and influencing both innate and adaptive immune responses involving DCs. Although CC10 has been suggested to exert immunomodulatory functions through interaction with Formyl Peptide Receptor-Like 1 (FPRL1) [54] or Integrin-α4β1 (VLA-4) [55], the specific receptor for CC10 on DCs is yet to be identified. Further research is essential to clarify the precise receptor through which CC10 exerts its effects on DCs and to deepen our comprehension of its immunomodulatory mechanisms.

Although our study and others have shown that CC10 does not act directly on T cells to affect T cell immune responses, our further investigation using a MLR assay, in which CD4^+^ T cells from OT-II mice were co-cultured with OVA_323-339_ peptide-pulsed DCs, revealed that CC10 treatment significantly inhibited the expansion of CD4^+^ T cells and the expression of activation markers CD25 and CD69 on CD4^+^ T cells. Additionally, CC10 impeded the differentiation of Th2 and Th17 cells, and suppressed the expression levels of inflammatory cytokines such as IL-2, IL-6, IL-13, IL-17A, and IFN-γ in the coculture system. These findings, along with our existing data illustrating the extensive regulatory effects of CC10 on the immunological activities, including differentiation, maturation, cytokine secretion, and endocytosis, of lung DCs in asthmatic mice as well as BMDCs in vitro, lead us to propose that CC10 inhibits allergen-induced Th2-type airway inflammatory responses in asthma primarily through modulating DC functions. Notably, a separate study has demonstrated that CC10 regulates Th17 cell differentiation and activation through DCs in the context of allergic rhinitis [31], similarly highlighting the crucial role of DCs in the immunomodulatory function of CC10. Taken together, DCs may represent crucial target regulatory cells of CC10 in chronic airway inflammatory diseases, playing a cardinal role in the process of CC10 dampening Th cell mediated inflammatory responses. The intricate interplay between CC10 and DCs emerges as a focal point in understanding and potentially manipulating immune responses in asthma and related diseases.

In conclusion, our study confirms reduced CC10 expression in asthma, correlating with higher IgE and lymphocyte levels. CC10 deficiency in mice intensifies HDM-induced allergic airway inflammation, while CC10 treatment reduces Th2 cytokines, inflammatory cell infiltration, and serum antigen-specific IgE levels, thereby reducing AHR, highlighting its vital role in curbing Th2-type inflammation in airways. Despite not directly inhibiting T cell activation, CC10 significantly modulates CD11b^+^CD103^−^ cDC2 subsets and immune activation function of lung DCs in asthma. MLR assays reveal that DCs are key for inhibitory effect of CC10 on CD4^+^ Th cell responses. Our findings demonstrate that CC10 indirectly attenuates Th2 cell-mediated airway inflammation by modulating the phenotype and function of lung DCs. This highlights the therapeutic potential CC10 in treating allergic asthma and possibly other related inflammatory airway diseases.

### Supplementary Information

Below is the link to the electronic supplementary material.

Supplementary file1 (DOCX 25 KB)

Supplementary file2 (DOCX 6923 KB)

## Data Availability

All the data are included in either the manuscript or in the supplementary information and are available from the corresponding author upon reasonable request.

## Abbreviations

HDM: House dust mite
AHR: Airway hyperresponsiveness
BALF: Bronchoalveolar lavage fluid
DC: Dendritic cell
BMDC: Bone marrow-derived DC
imDC: Immature DC
LPS: Lipopolysaccharides
CFSE: Carboxyfluorescein succinimidyl ester
OVA: Ovalbumin
MLR: Mixed lymphocyte reaction
WT: Wild type
