# Supplementary material for: Clara cell 10 (CC10) protein attenuates allergic airway inflammation by modulating lung dendritic cell functions
Source: Cell Mol Life Sci. 2024 Jul 30;81(1):321. doi: 10.1007/s00018-024-05368-z (PMC11335244; doi:10.1007/s00018-024-05368-z)
Supplement: Supplementary file 1 — Supplementary file1 (DOCX 25 KB) [file 18_2024_5368_MOESM1_ESM.docx]

**Clara cell 10 (CC10) protein** **attenuates allergic airway inflammation by** **modulating lung dendritic cell functions**

Yu-Dong Xu, Mi Cheng, Jun-Xia Mao, Xue Zhang, Pan-Pan Shang, Jie Long, Yan-Jiao Chen, Yu Wang, Lei-Miao Yin, Yong-Qing Yang*

**Supplementary Tables 1-3**

**Table S1. The detailed information of assay kits used in this study for ELISA and multiplex immunoassays**

| **ELISA Assay Kit** | **Source** | **Identifier** | **Lower Limit of**  **Quantification** | **Upper Limit of**  **Quantification** | **Sensitivity** |
| --- | --- | --- | --- | --- | --- |
| Mouse IL-4 | Biolegend | 431101 | 2 pg/mL | 125 pg/mL | - |
| Mouse IL-5 | Biolegend | 431201 | 7.8 pg/mL | 500 pg/mL | - |
| Mouse IL-6 | Biolegend | 431301 | 7.8 pg/mL | 500 pg/mL | - |
| Mouse IL-13 | Invitrogen | 88-7137 | 4 pg/mL | 500 pg/mL | 4.0 pg/mL |
| Mouse 23-Plex IL-2 | Bio-Rad | M60009RDPD | 3.72 pg/mL | 51,857 pg/mL | 0.6 pg/mL |
| Mouse 23-Plex IL-6 | Bio-Rad | M60009RDPD | 0.74 pg/mL | 12,053 pg/mL | 0.2 pg/mL |
| Mouse 23-Plex IL-10 | Bio-Rad | M60009RDPD | 2.95 pg/mL | 12,066 pg/mL | 1.0 pg/mL |
| Mouse 23-Plex IL-12 | Bio-Rad | M60009RDPD | 1.53 pg/mL | 25,024 pg/mL | 0.4 pg/mL |
| Mouse 23-Plex IL-13 | Bio-Rad | M60009RDPD | 47.2 pg/mL | 57,011 pg/mL | 38.7 pg/mL |
| Mouse 23-Plex IL-17A | Bio-Rad | M60009RDPD | 2.65 pg/mL | 43,337 pg/mL | 0.8 pg/mL |
| Mouse 23-Plex TNF-α | Bio-Rad | M60009RDPD | 5.8 pg/mL | 59,626 pg/mL | 1.4 pg/mL |
| Mouse 23-Plex IFN-γ | Bio-Rad | M60009RDPD | 1.84 pg/mL | 30,164 pg/mL | 1.2 pg/mL |
| Mouse 23-Plex G-CSF | Bio-Rad | M60009RDPD | 5.1 pg/mL | 84,244 pg/mL | 0.6 pg/mL |
| Mouse 23-Plex CXCL1 | Bio-Rad | M60009RDPD | 3.2 pg/mL | 18,202 pg/mL | 0.3 pg/mL |
| Mouse 23-Plex CCL5 | Bio-Rad | M60009RDPD | 2.78 pg/mL | 8,759 pg/mL | 0.6 pg/mL |

**Table S2. The detailed information of the FACS antibodies used in this study**

| **Antibodies** | **Clone** | **Dilution** | **Source** | **Identifier** |
| --- | --- | --- | --- | --- |
| PE-Cyanine7 anti-mouse CD25 | PC61 | 1: 200 | BD Biosciences | 552880 |
| FITC anti-mouse I-A/I-E | 2G9 | 1: 250 | BD Biosciences | 553623 |
| BUV 395 anti-mouse CD11c | HL3 | 1: 200 | BD Biosciences | 564080 |
| PerCP/Cyanine5.5 anti-mouse CD11b | M1/70 | 1: 200 | BD Biosciences | 101228 |
| Alexa Fluor 647 anti-mouse CCR7 | 4B12 | 1: 200 | BD Biosciences | 560766 |
| APC anti-mouse CD40 | 3/23 | 1: 200 | BD Biosciences | 558695 |
| FITC anti-Mouse CD3 | 17A2 | 1: 400 | BD Biosciences | 561798 |
| PE-Cyanine7 anti-mouse IL-4 | 11B11 | 1: 200 | BD Biosciences | 560699 |
| PE anti-Mouse IL-17A | TC11-18H10 | 1: 200 | BD Biosciences | 559502 |
| APC anti-mouse CD4 | RM4-5 | 1: 200 | BD Biosciences | 553051 |
| APC anti-mouse CD69 | H1.2F3 | 1: 200 | BD Biosciences | 560689 |
| Brilliant Violet 421 anti-mouse CD86 | GL-1 | 1: 200 | Biolegend | 105032 |
| PE anti-mouse CD103 | 2E7 | 1: 200 | Biolegend | 121406 |
| APC anti-mouse CD28 | 37.51 | 1: 200 | Biolegend | 102110 |
| Brilliant Violet 510 anti-mouse CD45 | 30-F11 | 1: 200 | Biolegend | 103138 |
| PerCP/Cyanine5.5 anti-mouse CD45 | 30-F11 | 1: 200 | eBioscience | 45-0451-82 |

**Table S3.** **Primer sequences for RT-qPCR in this study**

| Gene | Primer sequence | Product size (bp) |
| --- | --- | --- |
| *Il6* | Forward 5’ TGGTGACAACCACGGCCTTC 3’ | 104 |
|  | Reverse 5’ GCCTCCGACTTGTGAAGTGGT 3’ |  |
| *Tnfa* | Forward 5’ ACCCTCACACTCACAAACCAC 3’ | 212 |
|  | Reverse 5’ ATAGCAAATCGGCTGACGGT 3’ |  |
| *Il12* | Forward 5’ CACACTGGACCAAAGGGACT 3’ | 152 |
|  | Reverse 5’ TGATGAAGAAGCTGGTGCTG 3’ |  |
| *Tgfb1* | Forward 5’ AGGTCACCCGCGTGCTAATG 3’ | 258 |
|  | Reverse 5’ GCGTATCAGTGGGGGTCAGC 3’ |  |
| *Muc5ac* | Forward 5’ CCACTTTCTCCTTCTCCACACC 3’ | 119 |
|  | Reverse 5’ GGTTGTCGATGCAGCCTTGCTT 3’ |  |
| *Gapdh* | Forward 5’ TGCCCAGAACATCATCCCT 3’ | 233 |
|  | Reverse 5’ GGTCCTCAGTGTAGCCCAAG 3’ |  |
