# Supplementary material for: Clara cell 10 (CC10) protein attenuates allergic airway inflammation by modulating lung dendritic cell functions
Source: Cell Mol Life Sci. 2024 Jul 30;81(1):321. doi: 10.1007/s00018-024-05368-z (PMC11335244; doi:10.1007/s00018-024-05368-z)
Supplement: Supplementary file 2 — Supplementary file2 (DOCX 6923 KB) [file 18_2024_5368_MOESM2_ESM.docx]

**Clara cell 10 (CC10) protein** **attenuates allergic airway inflammation by** **modulating lung dendritic cell functions**

Yu-Dong Xu, Mi Cheng, Jun-Xia Mao, Xue Zhang, Pan-Pan Shang, Jie Long, Yan-Jiao Chen, Yu Wang, Lei-Miao Yin, Yong-Qing Yang*

**Supplemental Figures 1-5 and Figure legends**

**Supplementary Figure 1**


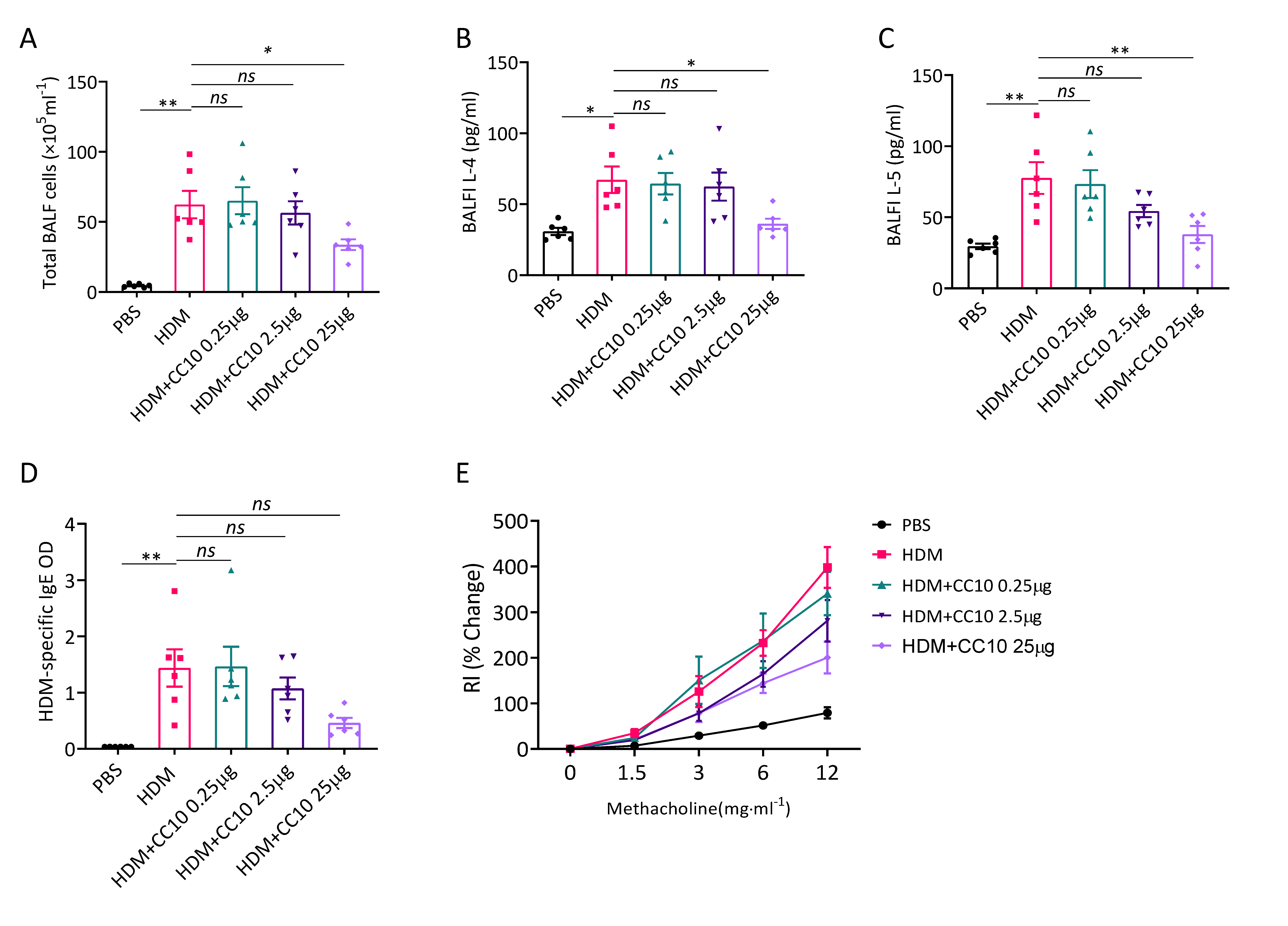


**Figure S1.** **Effects of different doses of CC10 administration on HDM-induced allergic asthma.** Mice were treated as illustrated in Fig. 2A, and CC10 was administrated at the dose of 0.25 μg, 2.5μg, and 25 μg before each sensitization and challenge. **(A)** Total leukocyte counts in the BALF from the indicated groups (*n* = 6 mice / group). **(B, C)** The levels of IL-4 (B) and IL-5 (C) in BALF as assessed by ELISA (*n* = 6 mice/group). **(D)** HDM-specific IgE levels were determined in mouse sera by means of ELISA (*n* = 6 mice/group). **(E)** Airway resistance in response to increasing doses of methacholine (Mch). Data were normalized to the baseline of each group (*n* = 6 mice/group). All data are shown as the mean ± SEM. (A-D) *P* values were calculated using one-way ANOVA followed by Games-Howell multiple-comparisons test. (E) Two-way ANOVA analysis was used. * *P* < 0.05, ** *P* < 0.01, ns = not significant.

**Supplementary Figure 2.**


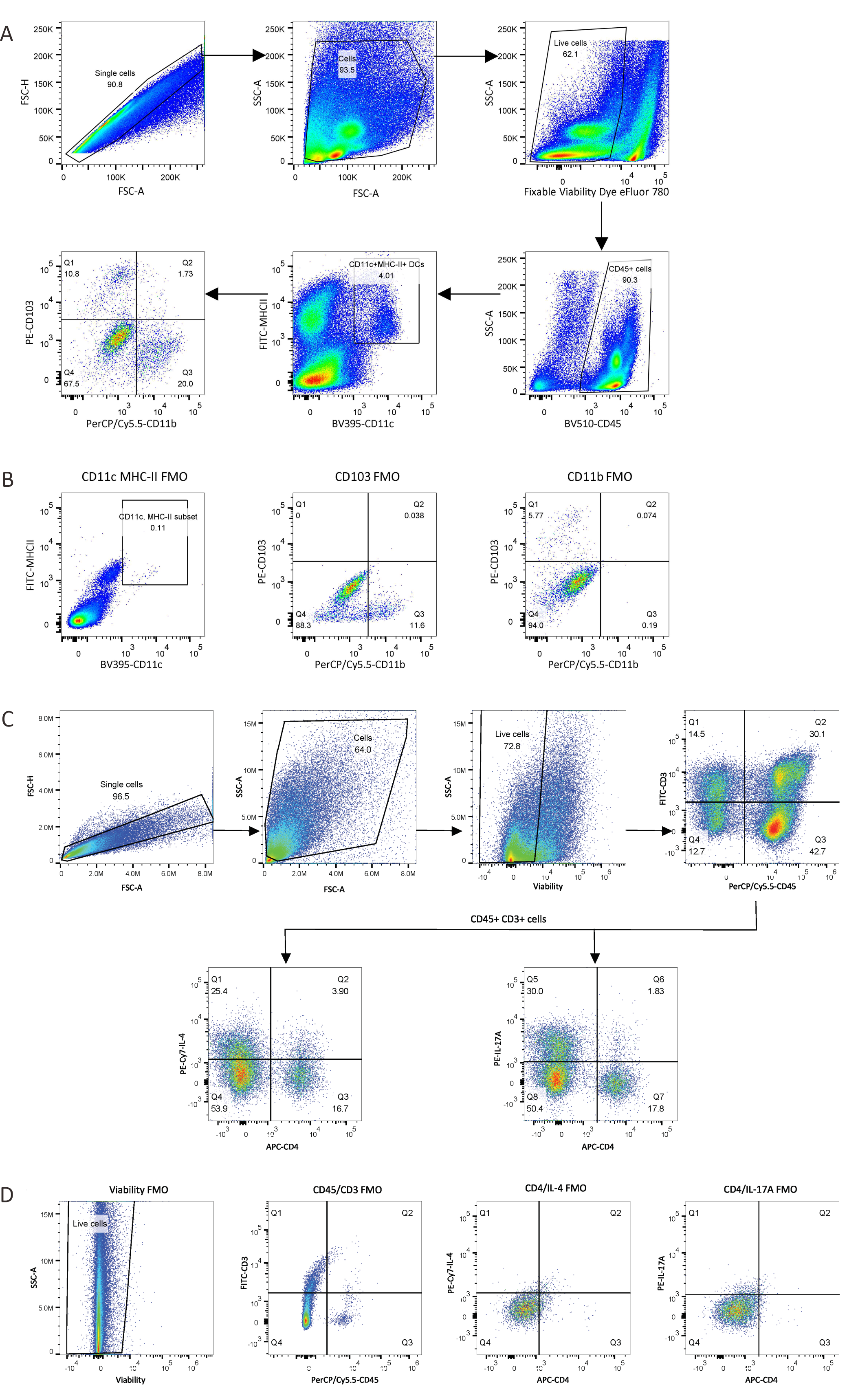


**Figure S2.** **Gating strategies for flow cytometric analyses.** **(A)** Flow cytometry gating scheme for lung DCs subsets. DCs were identified as CD11c^+^HLA-DR^+^ cells and were gated out of singlet live CD45^+^ cells. Gated DCs then analyzed by their expression of CD11b and CD103. **(B)** FMO (full antibody set minus one) controls used to set gates for the identification of DCs subsets. **(C)** Flow cytometry gating scheme for Th2 and Th17 cells. T cells were identified as CD3^+^CD45^+^ cells and were gated out of singlet live cells. Th2 and Th17 cells were further discriminated according to their intracellular marker expression, by which CD4^+^IL-4^+^ cells were considered as Th2 cells, and CD4^+^IL-17A^+^ cells considered as Th2 cells. **(D)** FMO controls used to set gates for the identification of Th2 and Th17 populations.

**Supplementary Figure 3**

**Figure S3. Effects of CC10 on the immune-activity of JAWS II cells.** **(A)** JAWS II cells were pre-treated with CC10 (1 μg/ml) or dexamethasone (Dex, 10 μM) and then stimulated with 200 ng/ml LPS for 24 h. CD86 expression on BMDCs was evaluated by flow cytometry. The graph shows averages from 5 independent experiments with similar results. **(B)** Endocytosis of JAWSII cells was measured by the cellular uptake of FITC-labeled dextran and quantified by flow cytometry. Representative histogram and quantification analysis from three independent experiments was shown. Data was normalized to PBS-treated control. All data are shown as the mean ± SEM. *P* values were calculated using one-way ANOVA followed by Games-Howell multiple-comparisons test. * *P* < 0.05, ** *P* < 0.01, ns = not significant.

**Supplementary Figure 4**


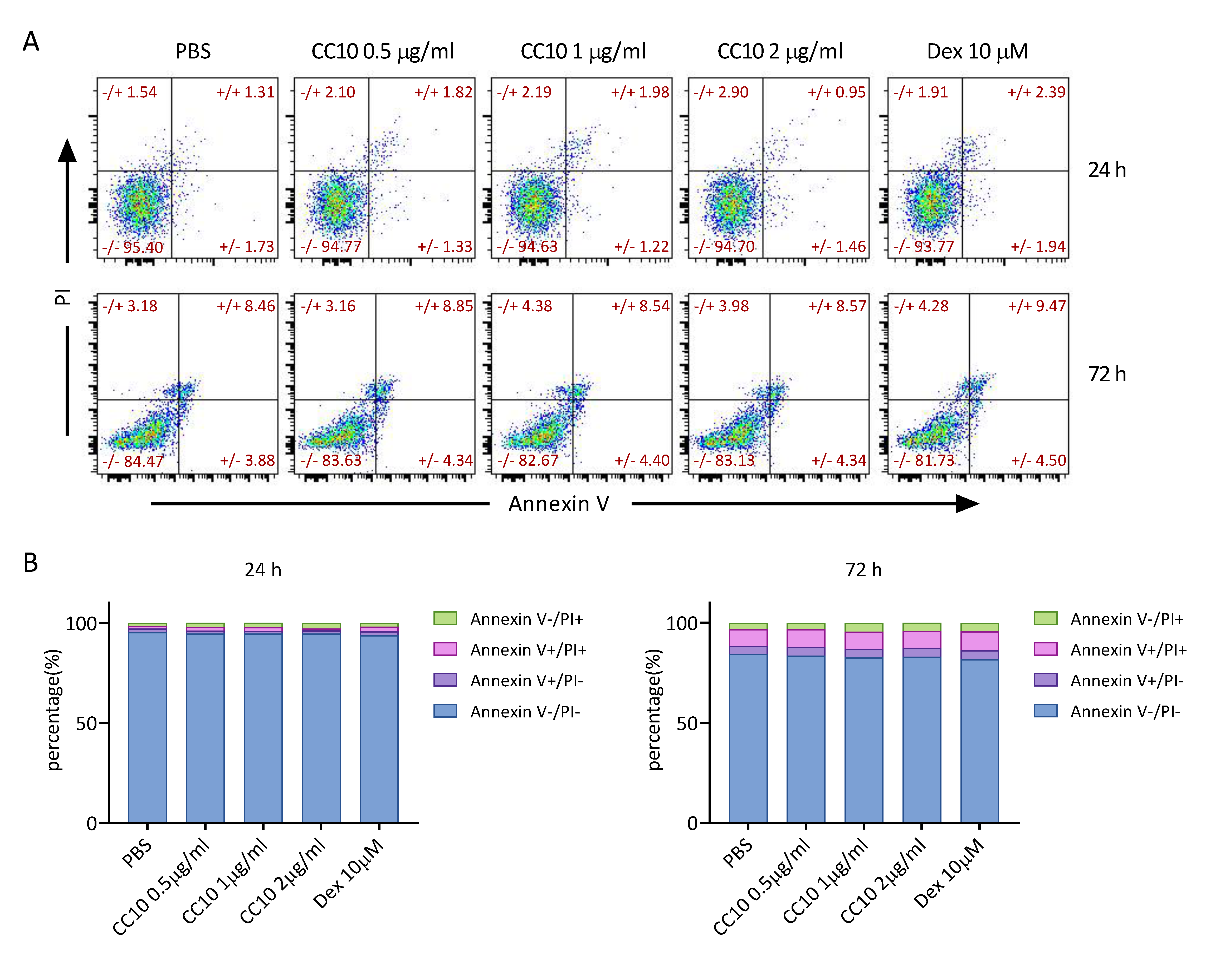


**Figure S4. Detection of cell viability in BMDCs by Annexin V-PI double-staining assay.** Cells treated with CC10 at different concentrations (0.5 μg/ml, 1 μg/ml, 2 μg/ml) or 10 μM Dex for 24 h and 72 h. **(A)** BMDCs were stained with FITC-Annexin V-PI and analyzed by flow cytometry. **(B)** Quantitative analysis of the percentages of viable cells (PI^-^/Annexin V^-^), necrotic cells (PI^+^/Annexin V^-^), early apoptotic cells (PI^-^/Annexin V^+^), and late apoptotic cells (PI^+^/Annexin V^+^) for each group at indicated time. Data were the averages from three independent experiments.

**Supplementary Figure 5**


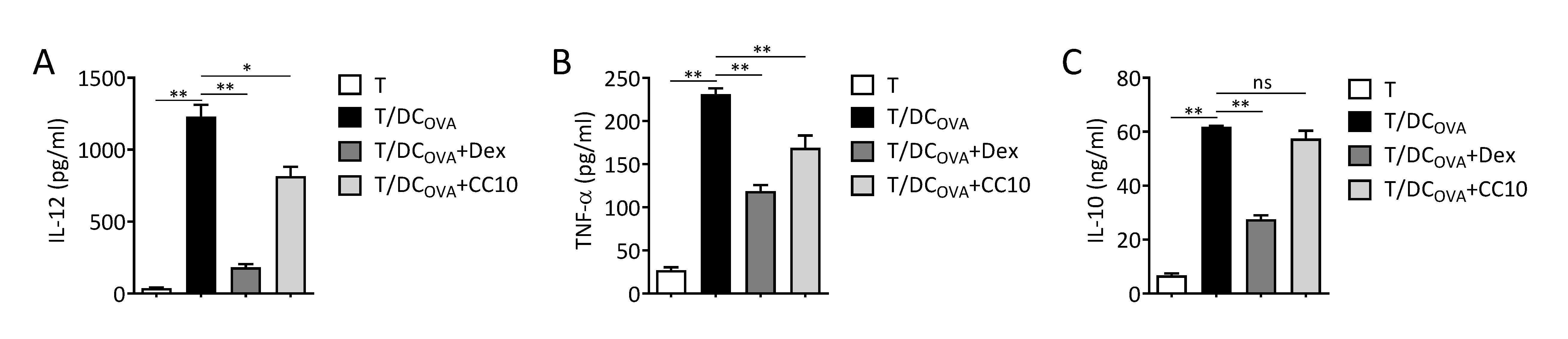


**Figure S5. CC10 regulated cytokine productions from T cells in the MLR culture.** The levels of IL-12 (A), TNF α (B), and IL-10 (C) in MLR culture supernatants were measured by ELISA (*n*= 4 per group). Data are presented as the mean ± SEM. *P* values were calculated using one-way ANOVA followed by Games-Howell multiple-comparisons test. * *P* < 0.05, ** *P* < 0.01, ns = not significant.
